# A multicenter phase 2 study of pomalidomide plus dexamethasone in patients with relapsed and refractory multiple myeloma: the Japanese MM-011 trial

**DOI:** 10.1186/s40164-016-0040-7

**Published:** 2016-04-18

**Authors:** Tatsuo Ichinohe, Yoshiaki Kuroda, Shinichiro Okamoto, Kosei Matsue, Shinsuke Iida, Kazutaka Sunami, Takuya Komeno, Kenshi Suzuki, Kiyoshi Ando, Masafumi Taniwaki, Kensei Tobinai, Takaaki Chou, Hitomi Kaneko, Hiromi Iwasaki, Chie Uemura, Hiromi Tamakoshi, Mohamed H. Zaki, Thomas Doerr, Shotaro Hagiwara

**Affiliations:** 1 Department of Hematology and Oncology, Research Institute for Radiation Biology and Medicine, Hiroshima University, 1-2-3 Kasumi, Minami-Ku, Hiroshima 734-8553 Japan; 2 Key University Hospital, 35 Shinanomachi, Shinjuku-Ku, Tokyo 160-8582 Japan; 3 Kameda Medical Center, 929 Higashi-chou, Kamogawa, Chiba 296-8602 Japan; 4 Nagoya City University Hospital, 1 Kawasumi, Mizuho-chou, Mizuho-Ku, Nagoya 467-8601 Japan; 5 Okayama Medical Center, 1711-1, Tamasu, Kita-Ku, Okayama 701-1154 Japan; 6 Mito Medical Center, 280 Sakuranosato, Ibarakimachi, Higashi-Ibaraki-Gun, Ibaraki 311-3193 Japan; 7 Japanese Red Cross Medical Center, 4-1-22 Hiroo, Shibuya-Ku, Tokyo 150-8935 Japan; 8 Tokai University Hospital, 143 Shimokasuya, Isehara, Kanagawa 259-1193 Japan; 9 University Hospital Kyoto Prefectural University of Medicine, Kawaramachi-Hirokoji, Kamigyo-Ku, Kyoto 602-8566 Japan; 10 National Cancer Center Hospital, 5-1-1 Tsukiji, Chuo-Ku, Tokyo 104-0045 Japan; 11 Niigata Cancer Center Hospital, 2-15-3 Kawagishimachi, Chuo-Ku, Niigata, 951-8133 Japan; 12 Osaka Red Cross Hospital, 5-30 Fudegasaki-cho, Tennouji-Ku, Osaka 543-8555 Japan; 13 Kyushu University Hospital, 3-1-1 Maidashi, Higashi-Ku, Fukuoka 812-8582 Japan; 14 Celgene, 1-21-1 Toyama, Shinjuku-Ku, Tokyo 162-8655 Japan; 15 Celgene Corporation, 86 Morris Avenue, Summit, NJ 07901 USA; 16 National Center for Global Health and Medicine Hospital, 15: 2-7-2 Marunouchi, Chiyoda-Ku, Tokyo 100-7010 Japan

**Keywords:** Relapsed/refractory multiple myeloma, Pomalidomide, Phase 2, Japan, Asian, Plasmacytoma

## Abstract

**Background:**

The immunomodulatory agent pomalidomide in combination with low-dose dexamethasone has demonstrated efficacy and safety for the treatment of relapsed and refractory multiple myeloma (RRMM) in phase 2 and 3 trials. However, these trials enrolled very few Asian patients.

**Methods:**

This phase 2 study investigated pomalidomide plus low-dose dexamethasone in 36 Japanese patients with RRMM after ≥2 prior therapies.

**Results:**

Patients enrolled in the study had a relatively high disease burden (81 % Durie–Salmon stage II or III) and were heavily pretreated (median, 6.5 prior antimyeloma regimens). The overall response rate was 42 % (1 patient with complete response and 14 with partial response), with an additional 44 % (16 patients) achieving stable disease (SD). Response rates in patients aged ≤65 years and >65 years were 47 and 35 %, respectively. None of the five patients with extramedullary disease achieved a response, with three of them maintaining SD of short duration. Median progression-free survival was 10.1 months after a 7.7-month median follow-up, and the median overall survival was not reached. The most frequent grade ≥3 adverse events (AEs) were neutropenia (64 %), anemia (42 %), and thrombocytopenia (31 %). The most frequent nonhematologic grade ≥3 AEs were pneumonia and decreased appetite (8 % each). Adverse events in patients aged >65 years were similar to those in patients aged ≤65 years, except for a higher rate of grade ≥3 pneumonia.

**Conclusions:**

Collectively, the results of this study demonstrate that pomalidomide plus low-dose dexamethasone is an effective and safe treatment for Japanese patients with RRMM, although careful attention needs to be paid to serious infections.

*Trial registration: Clinicaltrials.gov NCT02011113*

## Background

Although the introduction of thalidomide, lenalidomide, and bortezomib has improved the survival of patients with multiple myeloma (MM) [1], MM remains incurable, and median overall survival (OS) for patients who have become refractory to bortezomib and thalidomide or lenalidomide is only 9 months [2]. Pomalidomide is a distinct IMiD^®^ immunomodulatory compound with a mechanism of action that includes tumoricidal, immunomodulatory, and antiangiogenic effects [3]. In combination with low-dose dexamethasone, pomalidomide was approved in the United States, Canada, and the European Union for the treatment of patients with relapsed and refractory MM (RRMM) who have received ≥2 prior therapies, including lenalidomide and bortezomib, and who have demonstrated disease progression on the last therapy (United States, Canada, European Union) or within 60 days of completion of the last therapy (United States) [4–6]. In addition, this regimen has recently been approved for the treatment of Japanese patients with RRMM.

In a North American phase 1/2 RRMM study (MM-002), pomalidomide plus low-dose dexamethasone significantly extended progression-free survival (PFS) compared with pomalidomide alone [7, 8]. Furthermore, the regimen significantly improved both PFS and OS compared with high-dose dexamethasone alone in an international phase 3 trial (MM-003) [9]. The safety profile of pomalidomide plus low-dose dexamethasone was acceptable in both studies [8, 9]. However, the number of Asian patients who were enrolled in these previous studies was very small. Additionally, there are no phase 2 studies demonstrating the efficacy and safety profile of pomalidomide in Asian patients with RRMM.

Because pharmacokinetic (PK) and safety profiles of a drug can be affected by ethnicity [10–12], the phase 1 MM-004 study evaluated the tolerated dose, PK, safety, and efficacy of pomalidomide, alone and in combination with low-dose dexamethasone, in Japanese patients with RRMM [13]. Pomalidomide 4 mg/day, the recommended dose in the United States, Canada, and European Union [4–6], was identified as the tolerated dose in this patient population [13], consistent with previous observations in Caucasian patients [7]. These results led us to the phase 2 study, which investigated the efficacy and safety of pomalidomide plus low-dose dexamethasone in Japanese patients with RRMM.

## Results

### Patient characteristics

**Fig. 1:**
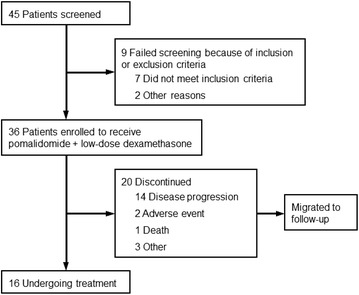
Patient screening, enrollment, and follow-up in the trial

A total of 36 patients were enrolled between December 2013 and July 2014 at 13 sites in Japan; all patients were of Asian origin (Fig. 1). The median age was 64.5 years, and 11 % of the patients were aged >75 years (Table 1). The median time from first diagnosis was 4.7 years. Five patients (14 %) presented with extramedullary plasmacytoma in bone (n = 4) and liver (n = 1). Patients had a relatively high disease burden, including Durie–Salmon stage II or III disease in 81 %, and were heavily pretreated, with a median of 6.5 prior antimyeloma regimens. All but 1 patient (97 %) were refractory to lenalidomide, and 58 % were refractory to both lenalidomide and bortezomib.

**Table 1:** Patient baseline characteristics

Characteristic	Pomalidomide plus dexamethasone (N = 36)
Age	
Median (range), years	64.5 (43–78)
>65 years, n (%)	17 (47.2)
>75 years, n (%)	4 (11.1)
Sex, n (%)	
Male	16 (44.4)
Female	20 (55.6)
Time from first diagnosis, median (range), years	4.7 (0.6–21.1)
ECOG performance status, n (%)	
0–1	33 (91.7)
2	3 (8.3)
Durie–Salmon stage, n (%)	
I	7 (19.4)
II	16 (44.4)
III	13 (36.1)
β_2_-microglobulin level, n (%)	
<3.5 mg/L	24 (66.7)
3.5–<5.5 mg/L	10 (27.8)
≥5.5 mg/L	2 (5.6)
Bone lesions, n (%)	22 (61.1)
Extramedullary plasmacytoma, n (%)	5 (13.9)
Creatinine clearance, n (%)	
<30 mL/min	0
30–<45 mL/min	0
45–<60 mL/min	8 (22.2)
60–<80 mL/min	13 (36.1)
≥80 mL/min	15 (41.7)
Prior antimyeloma therapies, median (range)	6.5 (2–15)
Prior stem cell transplant, n (%)	19 (52.8)
Prior therapies, n (%)	
Lenalidomide	36 (100.0)
Bortezomib	36 (100.0)
Thalidomide	12 (33.3)
Dexamethasone	35 (97.2)
Melphalan	31 (86.1)
Last prior therapy, n (%)	
Lenalidomide	21 (58.3)
Bortezomib	15 (41.7)
Refractory to prior therapies, n (%)	
Lenalidomide	35 (97.2)
Bortezomib	21 (58.3)
Both lenalidomide and bortezomib	21 (58.3)

*ECOG* Eastern Cooperative Oncology Group

### Study treatment

Median treatment duration was 5.5 months (range, 0.3–12.0 months), and the median number of treatment cycles was 6 (range, 1–13 cycles). At the data cutoff (February 3, 2015), 16 patients (44 %) remained on the protocol treatment. Disease progression was the most common reason for discontinuation (14 patients, 39 %). Three patients (8 %) discontinued because of an adverse event (AE), including 1 fatal AE of aggravated asthma and pneumonia, and three patients (8 %) discontinued for other reasons (Fig. 1).

### Efficacy

**Fig. 2:**
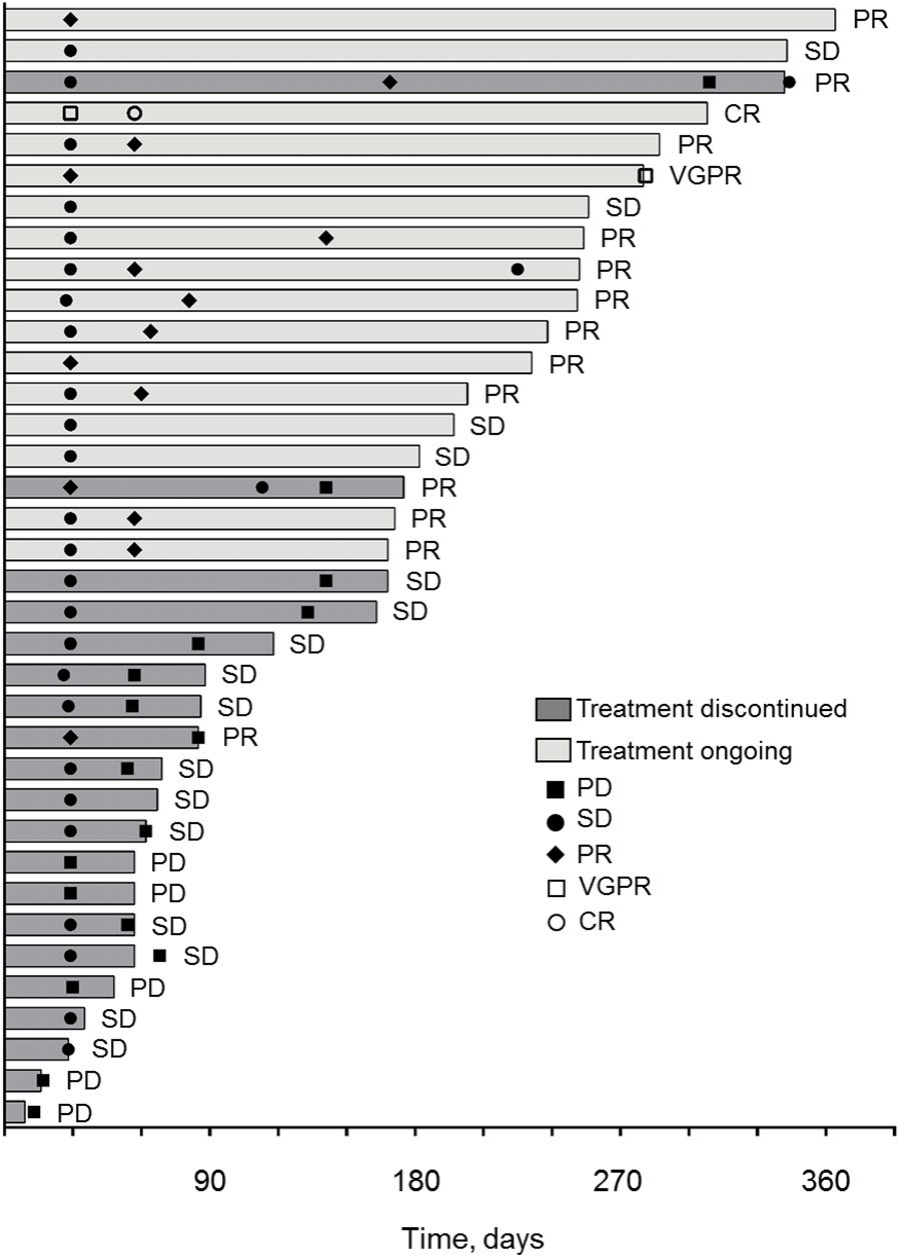
Treatment exposure and response duration of the enrolled patients. *CR* complete response, *PD* progressive disease, *PR* partial response, *SD* stable disease, *VGPR* very good partial response

**Fig. 3:**
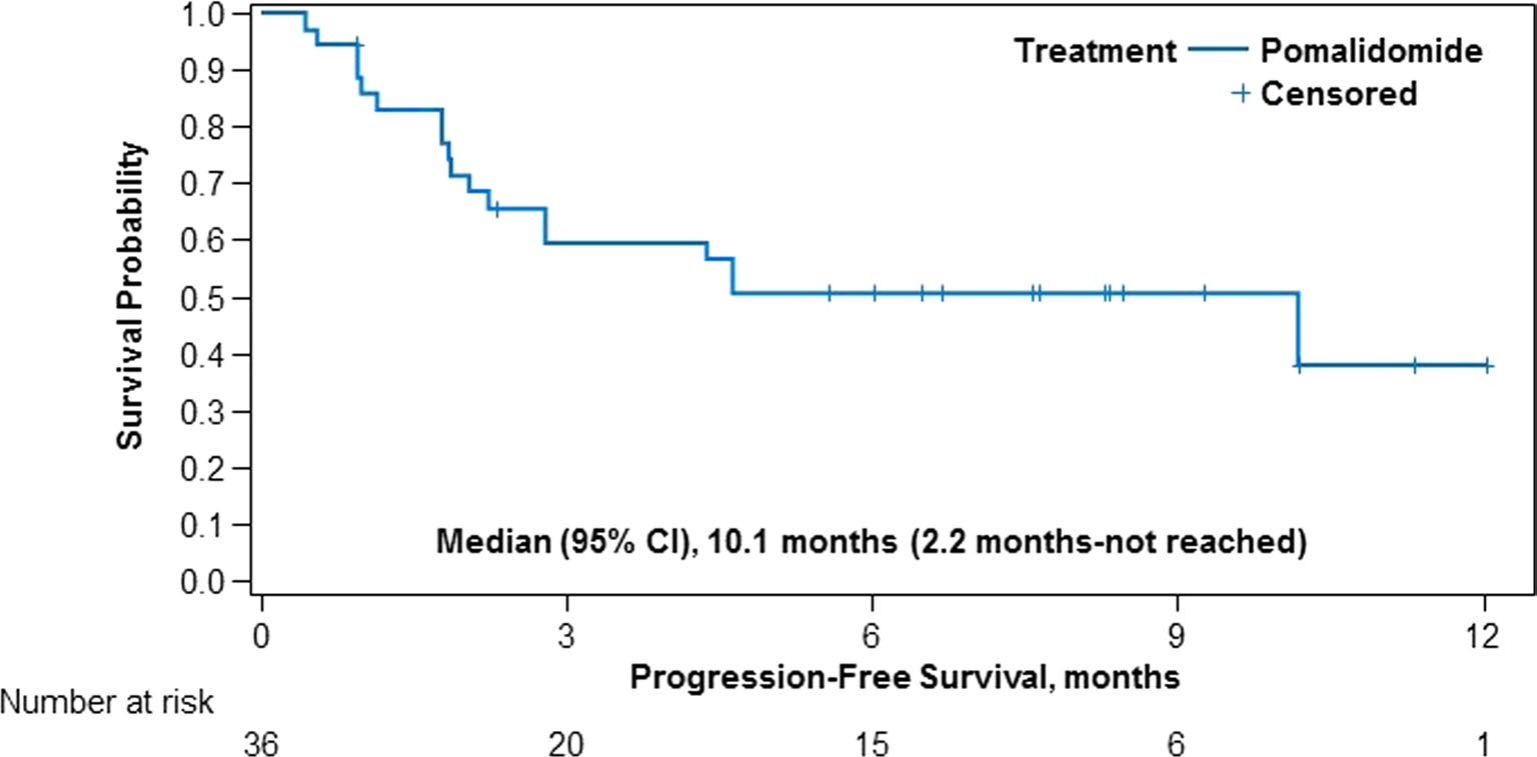
Kaplan–Meier estimates of progression-free survival from the start of first treatment to first documented disease progression or death, whichever occurred earlier, among patients who received pomalidomide plus low-dose dexamethasone in MM-011

All 36 patients received study treatment and were evaluable for efficacy. The overall response rate (ORR) was 42 % (15 patients; 95 % CI, 26–58 %), with 1 patient (3 %) achieving a complete response (CR) and 14 patients (39 %) achieving a partial response (PR; Table 2). Stable disease (SD) was recorded as the best response in 16 patients (44 %). Of these 36 evaluable patients, final pomalidomide doses at the last follow-up were 4 mg in 27 patiens, 3 mg in seven patients, and 2 mg in two patients with ORR of 44 % (12/27 patients, 1 CR and 11 PRs), 43 % (3/7 patients, all PRs) and 0 % in each dose group, respectively (Table 3). The median time to first response was 1.9 months, including 2 patients whose response improved from SD after ≥4 cycles of treatment (Fig. 2). The median duration of response (DOR) was not reached (95 % CI, 4.6 months-not estimable). After a median follow-up of 7.7 months, the median PFS was 10.1 months (Fig. 3). A prespecified final OS analysis was conducted using a data cutoff of September 25, 2015; after median follow-up of 11.3 months, the 1-year OS was 58.5 %.

**Table 2:** Responses based on IMWG criteria

Variable	Pomalidomide plus dexamethasone (N = 36)
Response rate, n (%)	
Overall response	15 (41.7)
Complete response	1 (2.8)
Very good partial response	0
Partial response	14 (38.9)
Stable disease	16 (44.4)
Progressive disease	5 (13.9)
Not evaluable	0
Time to response, median (range), months	1.9 (0.9–5.5)
Duration of response, median (range), months	Not reached (1.9–11.1)

*IMWG* International Myeloma Working Group

**Table 3:** Best response by final daily dose of pomalidomide

Variable	Final daily dose of pomalidomide^a^		
	4 mg (n = 27)	3 mg (n = 7)	2 mg (n = 2)
Best response rate, n (%)			
Overall response	12 (44.4)	3 (42.9)	0
Complete response	1 (3.7)	0	0
Very good partial response	0	0	0
Partial response	11 (40.7)	3 (42.9)	0
Stable disease	11 (40.7)	3 (42.9)	2 (100)
Progressive disease	4 (14.8)	1 (14.3)	0

^a^Daily dose as of February 3, 2015

In patients aged ≤65 years, the ORR was 47 % (9/19 patients, all PRs), and in patients aged >65 years, an ORR of 35 % was observed (6/17 patients, 1 CR and 5 PRs). One of four patients aged >75 years achieved a PR. Analysis of impact of disease stage at the time of protocol enrollment showed that ORR in Durie–Salmon stage III disease (23 %) tended to be lower than that in stage I (57 %) or stage II (50 %) disease, although it is not statistically significant (P = 0.28). ORR was 43 % among patients who were refractory to lenalidomide (15/35 patients, 1 CR and 14 PRs) and 33 % among those refractory to both lenalidomide and bortezomib (7/21 patients, all PRs). Recent studies have shown that thalidomide is effective in patients refractory to bortezomib and lenalidomide treatment [14, 15]. ORR among patients who had previously received thalidomide and those who did not receive thalidomide was 33 % (4/12 patients, all PRs) and 46 % (11/24 patients, with 1 CR and 10 PRs), respectively, with no significant difference between two groups (P = 0.72), although the latter showed a trend toward longer PFS (3.3 months versus not reached, P = 0.21). Median OS was not reached for patients who received or did not receive prior thalidomide. Of the five patients with plasmacytomas, none achieved a response, with SD recorded as the best response in three patients. The median PFS for these five patients was 1.8 months.

### Safety

All 36 patients reported ≥1 AE, and 31 patients (86 %) experienced a grade ≥3 AE, regardless of causality (Table 4). The most frequently reported grade ≥3 hematologic AEs regardless of causality were neutropenia (23 patients, 64 %), anemia (15 patients, 42 %), and thrombocytopenia (11 patients, 31 %). The most frequently reported grade ≥3 nonhematologic AEs regardless of causality were pneumonia (three patients, 8 %) and decreased appetite (three patients, 8 %). Other frequently reported AEs (any grade) were pyrexia (nine patients, 25 %); nasopharyngitis (eight patients, 22 %); and gastrointestinal disorders, including constipation (eight patients, 22 %), diarrhea, and nausea (seven patients, 19 % each).

**Table 4:** Summary of the most commonly reported adverse events (regardless of causality and reported at any grade in ≥10 % of patients)

n (%)	All patients (N = 36)		Age ≤65 years (n = 19)		Age >65 years (n = 17)	
	All grades	Grade ≥ 3	All grades	Grade ≥ 3	All Grades	Grade ≥ 3
Patients with ≥ 1 AE	36 (100.0)	31 (86.1)	19 (100.0)	16 (84.2)	17 (100.0)	15 (88.2)
Neutropenia	26 (72.2)	23 (63.9)	16 (84.2)	14 (73.7)	10 (58.8)	9 (52.9)
Anemia	17 (47.2)	15 (41.7)	9 (47.4)	8 (42.1)	8 (47.1)	7 (41.2)
Thrombocytopenia	17 (47.2)	11 (30.6)	11 (57.9)	6 (31.6)	6 (35.3)	5 (29.4)
Pyrexia	9 (25.0)	0	5 (26.3)	0	4 (23.5)	0
Constipation	8 (22.2)	2 (5.6)	6 (31.6)	1 (5.3)	2 (11.8)	1 (5.9)
Nasopharyngitis	8 (22.2)	0	2 (10.5)	0	6 (35.3)	0
Lymphopenia	7 (19.4)	6 (16.7)	4 (21.1)	3 (15.8)	3 (17.6)	3 (17.6)
Diarrhea	7 (19.4)	0	5 (26.3)	0	2 (11.8)	0
Nausea	7 (19.4)	0	4 (21.1)	0	3 (17.6)	0
Leukopenia	6 (16.7)	4 (11.1)	3 (15.8)	2 (10.5)	3 (17.6)	2 (11.8)
Peripheral edema	6 (16.7)	0	2 (10.5)	0	4 (23.5)	0
Rash	6 (16.7)	0	4 (21.1)	0	2 (11.8)	0
Insomnia	6 (16.7)	0	2 (10.5)	0	4 (23.5)	0
Pneumonia	5 (13.9)	3 (8.3)	1 (5.3)	0	4 (23.5)	3 (17.6)
Decreased appetite	5 (13.9)	3 (8.3)	2 (10.5)	1 (5.3)	3 (17.6)	2 (11.8)
Malaise	5 (13.9)	0	4 (21.1)	0	1 (5.9)	0
Dysgeusia	5 (13.9)	0	2 (10.5)	0	3 (17.6)	0
Hypoxia	4 (11.1)	1 (2.8)	3 (15.8)	1 (5.3)	1 (5.9)	0
Epistaxis	4 (11.1)	0	2 (10.5)	0	2 (11.8)	0
Upper respiratory tract infection	4 (11.1)	0	2 (10.5)	0	2 (11.8)	0

*AE* adverse event

Peripheral neuropathy (PN) of any grade occurred in three patients (8 %) and was considered to be treatment-related in all cases. No occurrences of deep vein thrombosis or pulmonary embolism were reported; all patients received thromboprophylaxis, most commonly with aspirin (94 % of patients). Febrile neutropenia was observed in one patient (3 %), and severe infections and infestations occurred in three patients (8 %). Serious AEs were reported in 13 patients (36 %) and were considered treatment related in six patients (17 %). Constipation and pneumonia were the only 2 treatment-related serious AEs that occurred in >1 patient (two patients, 6 % each).

Pomalidomide dosing was interrupted in 15 patients (42 %) and reduced in nine patients (25 %) due to AEs. AEs leading to dose reductions in >1 patient were thrombocytopenia (three patients, 8 %), anemia (two patients, 6 %), and leukopenia (two patients, 6 %). Three patients (8 %) had ≥1 AE that led to discontinuation of study treatment, all of which were considered treatment related. AEs leading to discontinuation were asthma, dyspnea, pleural effusion, anemia, pyrexia, and pneumonia. Nine patients (25 %) died during the study; eight deaths were due to progression of MM, and one was due to an AE of pneumonia and aggravated asthma that was suspected to be related to study treatment.

The AE profile in patients aged >65 years was broadly consistent with that in patients aged ≤65 years (Table 4), except for a higher rate of grade ≥3 pneumonia in the older patients.

## Discussion

This study demonstrates that pomalidomide in combination with low-dose dexamethasone is an effective regimen that confers disease stabilization or regression in 86 % of heavily pretreated Japanese patients with RRMM, with an acceptable safety profile consistent with the prior studies in other regions. The phase 3 MM-003 trial, which was conducted in 93 centers in Europe, Russia, Australia, Canada, and the United States, previously investigated pomalidomide plus dexamethasone in 302 patients with RRMM who had received prior therapy with both lenalidomide and bortezomib [9]. MM-003 found that pomalidomide plus low-dose dexamethasone significantly improved PFS, OS, and ORR vs high-dose dexamethasone alone [9].

Due to the small sample size of MM-011, results may be less precise and the ability to compare with other trials such as MM-003 is limited, therefore findings in our study should be interpreted with caution. However, the results reported here, including an ORR of 42 % (compared with 31 % in MM-003), suggest that efficacy outcomes of pomalidomide-based salvage treatment in RRMM could be more favorable depending on the criteria used for patient selection [9]. It will be interesting to determine if, as in MM-003, patients with a greater response (either SD or ≥PR) experience a longer OS compared with patients with progressive disease [16]. Additionally, median PFS in MM-011 was substantially longer than in the MM-003 trial (10.2 vs 4.0 months, respectively) [9], probably reflecting the differences in background characteristics of the patients participating in these studies.

One possible explanation for the observed differences in outcomes between MM-011 and MM-003 is the longer duration of treatment in MM-011 (median, 5.5 vs 4.2 months) [9]. Subgroup analysis of MM-003 showed that some patients who achieved only SD within four cycles of treatment went on to improve their response status with continued treatment beyond four cycles [16]. Thus, the improved outcomes in MM-011 may reflect prolonged time on therapy.

Additionally, the observed differences in outcomes may reflect variability in disease characteristics between the patient population of MM-011 and the pomalidomide plus low-dose dexamethasone arm of MM-003. Several factors associated with poor outcomes were less common in MM-011 than in MM-003. For example, impaired renal function (creatinine clearance <60 mL/min) was present in 22 % of patients in MM-011 compared with 31 % of patients in MM-003 [9]. Renal function may reflect the disease status of MM; however, preliminary data from the MM-008 study showed that pomalidomide dosing need not be reduced in patients with renal function impairment [17]. Additionally, slightly fewer patients in MM-011 had bone lesions compared with MM-003 (61 vs 68 %) [9]. Advanced lytic lesions were reported to be a risk factor associated with poor survival in patients who receive pomalidomide [18]. Finally, patients had lower levels of serum β_2_-microglobulin in MM-011 vs MM-003 (Celgene Corporation, MM-003 clinical study report, unpublished observation). Higher serum β_2_-microgloblin has been identified as a risk factor associated with shorter OS in patients refractory to bortezomib and IMiD immunomodulatory agents [2].

Of note, three out of five patients with plasmacytomas in MM-011 achieved SD following treatment with pomalidomide plus low-dose dexamethasone, albeit with a short median PFS of 1.8 months. Extramedullary disease associated with MM is known for poor prognosis, even after the introduction of novel agents, including lenalidomide and bortezomib, and thalidomide [19, 20]. Therefore, improved treatment options are urgently needed for this patient population. Because pomalidomide has been shown to have more potent in vitro antimyeloma activity compared with conventional IMiD agents [21–23], further studies are needed to determine whether pomalidomide plus low-dose dexamethasone provides a survival benefit in patients with extramedullary disease or nonsolitary plasmacytoma.

Use of prior treatment options also notably differed between MM-011 and MM-003. Fewer patients in MM-011 had received a prior stem cell transplant than patients in MM-003 (53 vs 71 %) [9]. Although patients had received a higher number of prior antimyeloma therapies in MM-011 vs MM-003 (median, 6.5 vs 5 therapies), median time from initial diagnosis was shorter in MM-011 (4.7 vs 5.3 years). A similar proportion of patients in MM-011 and MM-003 were refractory to lenalidomide (97.2 vs 95 %). Fewer patients in MM-011 were refractory to bortezomib alone (58 vs 79 %) or to both lenalidomide and bortezomib (58 vs 75 %). The lower levels of bortezomib refractory disease in MM-011 may result from differences in eligibility criteria: MM-003, but not MM-011, included patients with primary refractory disease and required that patients had experienced prior treatment failure with both lenalidomide and bortezomib. These differences suggest that patients in MM-011 had disease that was not advanced as in MM-003, potentially accounting for the higher response rates and longer PFS observed. However, subanalysis of MM-003 found no effect of prior treatment on response rate [16].

The chromosomal aberrations del(17p) and t(4;14) are associated with adverse prognosis, with median event-free survival from diagnosis of only 20.6 months and 15 months, respectively [24–27]. Therefore, the efficacy of pomalidomide plus low-dose dexamethasone in MM-011 may have been affected by the proportion of patients with these poor-risk chromosomal aberrations. However, collection of data on chromosomal aberrations was not included in the MM-011 protocol, and this analysis is not available.

The non-hematologic AE profile in MM-011 was generally consistent with that of pomalidomide plus low-dose dexamethasone treatment in MM-003, with a few exceptions. The incidence of severe infections was lower in MM-011 (8.3 % with grade ≥3 infection or infestation) than in MM-003 (30 % with grade 3/4 infection), as was the incidence of any grade febrile neutropenia (3 vs 10 %) [9]. Venous thromboembolism (VTE) is associated with decreased survival in MM [28] and is a rare but potentially serious AE that has been reported with IMiD therapy [29, 30]. In a preliminary study in 1035 Japanese patients with MM treated with thalidomide, the incidence of VTE was found to be lower than that in Western patients [31], potentially associated with genetic background and other factors related to ethnicity [32, 33]. In prior studies, appropriate thromboprophylaxis has been selected based on the risk of VTE for Japanese patients. With appropriate protocol-mandated thromboprophylaxis in MM-011, no cases of VTE were reported. Finally, PN is a common and potentially treatment-limiting AE associated with thalidomide and bortezomib; however, pomalidomide as well as lenalidomide do not appear to cause substantial neurotoxicity [9, 34]. In MM-011, PN of any grade occurred in three patients (8 %) and did not lead to treatment discontinuation.

Grade ≥3 hematologic AEs occurred more frequently in MM-011 than in MM-003, including neutropenia (64 vs 48 %), anemia (42 vs 33 %), and thrombocytopenia (31 vs 22 %); however, the rate of all-grade hematologic AEs in MM-011 was similar to MM-003 [9]. In the Japanese MM-004 study, the incidence of grade ≥3 neutropenia was also higher than in MM-003 (67 vs 48 %) [9, 13]. This suggests that a greater number of Japanese patients may have a greater need for dose adjustments in response to hematologic AEs compared with those from other regions. The observed differences in AEs are not likely to be due to PK differences, as MM-004 found PK parameters of pomalidomide in Japanese patients with RRMM to be similar to those reported for pomalidomide in other RRMM populations, with limited accumulation after multiple doses [13]. Importantly, hematologic AEs were manageable with temporary discontinuation of treatment or with concomitant administration of granulocyte colony-stimulating factor. The successful management of AEs may have contributed to extended duration of treatment in MM-011.

## Conclusions

In conclusion, pomalidomide 4 mg/day has been confirmed as the acceptable starting dose for Japanese patients, with dexamethasone administered at a dose of 40 mg/day (reduced to 20 mg/day for patients aged >75 years). Pomalidomide plus low-dose dexamethasone is a relatively safe and highly efficacious treatment for Japanese patients with RRMM who have previously received both lenalidomide and bortezomib. Patients who achieve stable disease or better response while on pomalidomide can continue to benefit from this therapy. Additional studies may be required to further define those patients that would derive the most benefit from pomalidomide-based therapies.

## Methods

### Patients

Eligible patients had documented MM and relapsed and refractory disease, defined as disease progression after ≥SD for ≥1 cycle of treatment or during or within 60 days of completing treatment. Other inclusion criteria were ≥2 prior therapies (including ≥2 cycles of lenalidomide and bortezomib, separately or in combination), age ≥20 years, and Eastern Cooperative Oncology Group performance status ≤2. Exclusion criteria included previous pomalidomide treatment; hypersensitivity to thalidomide, lenalidomide, or dexamethasone; absolute neutrophil count <1000/μL; platelet count <75,000/μL (or <30,000/μL if ≥50 % of bone marrow nucleated cells were plasma cells); creatinine clearance <45 mL/min using the Cockcroft-Gault formula; corrected serum calcium >14 mg/dL (>3.5 mmol/L); hemoglobin <8 g/dL (<4.9 mmol/L); liver enzyme concentrations >3.0× upper limit of normal (ULN); total bilirubin >2.0 mg/dL (34.2 μmol/L; or ≥3.0× ULN for hereditary benign hyperbilirubinemia); congestive heart failure (New York Heart Association Class III/IV); myocardial infarction within 12 months; unstable or poorly controlled angina pectoris; and PN grade ≥2.

All patients provided informed consent; the study was approved by each study site’s institutional review board and was conducted in accordance with the Declaration of Helsinki and the International Conference on Harmonisation guidelines on good clinical practice. The trial is registered as clinicaltrials.gov identifier NCT02011113.

### Study design

MM-011 was a phase 2 multicenter, single-arm, open-label study conducted in Japan (Fig. 1). Patients received pomalidomide (4 mg/day orally, days 1-21, 28-day cycles) and dexamethasone (40 mg/day [20 mg/day if aged >75 years] orally, days 1, 8, 15, and 22), consistent with United States and European Union approved dosing [4, 5]. Treatment was continued until disease progression, unacceptable toxicity, or withdrawal. All patients received thromboprophylaxis with low-dose aspirin, low-molecular-weight heparin, or equivalent.

Pomalidomide was interrupted for grade 4 neutropenia or thrombocytopenia, grade ≥3 constipation, VTE, rash, PN, or other pomalidomide-related AE, or grade ≥2 hypothyroidism or hyperthyroidism. Additionally, pomalidomide was interrupted for febrile neutropenia (any grade). Pomalidomide could be restarted at the same level or decreased by 1 mg. Discontinuation of pomalidomide was indicated for rash (grade 4 or blistering) or grade ≥4 PN. Dexamethasone dose was modified for grade ≥3 edema, hyperglycemia, or any other dexamethasone-related AE. Additionally, dexamethasone was modified for grade ≥2 confusion/mood alteration or muscle weakness, or any grade dyspepsia. Dexamethasone was discontinued for acute pancreatitis.

The primary endpoint was response rate according to the International Myeloma Working Group (IMWG) criteria [35]. Enrollment of 37 patients was planned using the expected response rate of 25 % based on the efficacy evaluable population, the threshold response rate of 10 %, on one-sided alpha of 0.05 and the statistical power of 80 % based on the test for one sample proportion. Secondary endpoints included response rate according to European Group for Blood and Marrow Transplantation (EBMT) criteria [36], time to response (TTR), DOR, PFS, and safety.

### Efficacy assessments

Response was assessed by investigators using IMWG criteria and was confirmed by the members of an independent response adjudication committee, who also confirmed responses using EBMT criteria. TTR was calculated as the time from first dose to first documented response. DOR was defined as the time from first documented response to first documented disease progression. PFS was the time from first dose to first documented disease progression or death, whichever occurred earlier, and was estimated using the Kaplan–Meier method.

### Safety evaluation

AEs were graded according to the National Cancer Institute Common Terminology Criteria for Adverse Events version 4.0 [37] throughout treatment and for 28 days after last dose. Other safety assessments included VTE monitoring, physical examinations, vital signs, electrocardiograms, and standard clinical laboratory assessments (thyroid function, hematology, serum chemistry, urinalysis, creatinine clearance, and virology).

## Abbreviations

AE: adverse event
CR: complete response
DOR: duration of response
EBMT: European Group for Blood and Marrow Transplantation
ECOG: Eastern Cooperative Oncology Group
IMWG: International Myeloma Working Group
ITT: intent to treat
MM: multiple myeloma
ORR: overall response rate
OS: overall survival
PFS: progression-free survival
PK: pharmacokinetics
PN: peripheral neuropathy
PR: partial response
RRMM: relapsed and refractory MM
SD: stable disease
TTR: time to response
ULN: upper limit of normal
VGPR: very good partial response
VTE: venous thromboembolism
